# Dipeptidyl Peptidase-4 Inhibitory Activity of Buckwheat Flour-Derived Peptides and Oral Glucose Tolerance Test of Buckwheat Flour Hydrolysates in Rats

**DOI:** 10.3390/foods15010092

**Published:** 2025-12-29

**Authors:** Noe Mitsui, Kouji Shiono, Yoshiya Seto, Tadasu Furusho, Chika Saito, Kosaku Takahashi

**Affiliations:** 1 Faculty of Applied Biosciences, Tokyo University of Agriculture, 1-1-1 Sakuragaoka, Setagaya-ku, Tokyo 156-8502, Japancs208491@nodai.ac.jp (C.S.); 2 School of Agriculture, Meiji University, 1-1-1 Higashi-Mita, Tama-ku, Kawasaki 214-8571, Kanagawa, Japan; yoshiya@meiji.ac.jp; 3 Faculty of International Agriculture Food Studies, Tokyo University of Agriculture, 1-1-1 Sakuragaoka, Setagaya-ku, Tokyo 156-8502, Japan; tfurusho@nodai.ac.jp

**Keywords:** peptide, dipeptidyl peptidase-4, inhibition, diabetes, buckwheat

## Abstract

Dipeptidyl peptidase-4 (DPP-4) is a protease that degrades incretin and inhibits the secretion of insulin. Consequently, DPP-4 inhibition promotes insulin secretion and prevents the onset of type 2 diabetes. Given the growing interest in food-derived DPP-4 inhibitory peptides as potential functional ingredients, buckwheat (*Fagopyrum esculentum*) represents a promising source; however, few studies have investigated the bioactivity of peptides derived from buckwheat flour hydrolysates. In this study, two DPP-4 inhibitory peptides, Ile-Pro-Trp and Ile-Pro-Leu, were identified through purification of buckwheat flour hydrolysate and liquid chromatography–tandem mass spectrometry analysis. In a rat oral glucose tolerance test (OGTT), a fraction of buckwheat flour hydrolysate, crudely purified by reverse-phase column chromatography, showed a non-significant trend toward reducing increases in blood glucose. To our knowledge, this study is the first to show that Ile-Pro-Trp isolated from food protein hydrolysates exhibits considerable DPP-4 inhibitory activity. Moreover, this is the first study identifying Ile-Pro-Ile as a DPP-4 inhibitor from a plant source.

## 1. Introduction

Diabetes mellitus is one of the most serious and rapidly increasing diseases worldwide, and it poses a major threat to global public health. According to the International Diabetes Federation, an estimated 588.7 million adults will be living with diabetes in 2024, corresponding to a global prevalence of 11.1%, and the number of adults living with diabetes is expected to rise to 853 million by 2050 [1]. Diabetes is broadly categorized into two main types: type 1 diabetes, caused primarily by autoimmune destruction of pancreatic β-cells, and type 2 diabetes, which accounts for more than 90% of cases. Type 2 diabetes develops mainly because of impaired insulin action, insufficient insulin secretion, or a combination of both. Among the various mechanisms contributing to this disease, a key factor is the rapid degradation of incretin hormones, which stimulate insulin secretion after food intake. This degradation is catalyzed by dipeptidyl peptidase-4 (DPP-4) [2].

DPP-4 is a serine protease that specifically cleaves peptides when proline or valine residues occupy the second position from the N-terminus. Incretins, such as glucagon-like peptide-1 (GLP-1) and glucose-dependent insulinotropic polypeptide (GIP) are typical DPP-4 substrates. Once cleaved and inactivated, these hormones lose their ability to enhance insulin secretion, which results in impaired postprandial glucose regulation. Consequently, inhibition of DPP-4 prevents incretin inactivation, prolongs its biological activity, and contributes to improved glucose homeostasis. Thus, DPP-4 inhibition has become a well-established strategy for the prevention and treatment of diabetes, and DPP-4 inhibitors are widely used as antidiabetic drugs [3].

In addition to pharmacological approaches, there is a growing demand for functional foods that help maintain health and prevent disease. This trend is driven by increasing health awareness and a global emphasis on preventive healthcare. Foods containing bioactive components that modulate metabolic processes are being actively developed. Among these, peptides generated through the enzymatic hydrolysis of dietary proteins have attracted considerable attention because of their diverse physiological activities [4]. For example, protease hydrolysates derived from sardines have been explored as functional ingredients capable of inhibiting angiotensin I-converting enzyme (ACE), thereby regulating blood pressure [5,6,7]. Similarly, an increasing number of studies have reported that natural food-derived peptides inhibit DPP-4 activity. DPP-4 inhibitory peptides have been identified in hydrolysates of fish skin, walnut, and milk proteins, suggesting that food-derived peptides may serve as promising natural agents for glucose regulation and diabetes prevention [8,9,10].

Buckwheat (*Fagopyrum esculentum*), a pseudocereal of the Polygonaceae family, has long been valued as a nutrient-rich food source, and it is cultivated extensively across the world [11,12]. Its seeds and flour are rich in starch, proteins, lipids, dietary fiber, vitamins, and minerals and are used in a variety of traditional and modern foods. Buckwheat also contains abundant phenolic compounds such as rutin and other polyphenols, which exhibit strong antioxidant activity and have been associated with health-promoting effects [13,14,15]. Despite its well-documented nutritional and functional properties, relatively few studies have examined its potential as a peptide source with DPP-4 inhibitory activity. Identifying such bioactive peptides from buckwheat would not only expand our understanding of their functional components, but also support the development of novel food products with antidiabetic potential.

In the present study, we investigated the DPP-4 inhibitory activity of peptides derived from buckwheat flour hydrolysates. Specifically, we sought to identify the active peptide sequences through enzymatic hydrolysis and biochemical analyses. Furthermore, we conducted an oral glucose tolerance test (OGTT) using a crude fraction of buckwheat flour hydrolysate.

## 2. Materials and Methods

### 2.1. Assay for DPP-4 Inhibitory Activity

DPP-4 inhibitory activity was measured as described by Sato et al. with some modifications [16]. Briefly, 20 µL of 0.3 mM Gly-Pro-MCA (Fujifilm Wako Pure Chemical Corporation, Osaka, Japan), 20 µL of sample solution, 150 µL of 50 mM Tris-HCl buffer (pH 7.5), and 10 µL of a recombinant human DPP-4 solution (0.5 mg/mL, 200 unit/mg, NKMAXBio, Seongnam-si, Republic of Korea) were added to each well of a 96-well black microtiter plate. Fluorescence intensity was measured every 15 min for 45 min using a dual-mode microplate reader (Infinite^®^ M Nano^+^, Tecan, Männedorf, Switzerland) at 37 °C; with an excitation wavelength of 360 nm and emission at 450 nm. A reaction mixture containing water instead of the sample solution served as a control. DPP-4 inhibitory activity (%) was calculated based on the fluorescence intensities of the samples and control. Subsequent purification steps were guided by the results of the assay.

### 2.2. Preparation of Buckwheat Flour Hydrolysate

Buckwheat flour (10 g, Masudaya shokuhin, Nagano, Japan) was added to 150 mL of 100 mM phosphate buffer (pH 8.0) and preheated at 50 °C. Subsequently, 500 µL each of Alcalase^®^ (Sigma-Aldrich, Burlington, MA, USA) and Flavourzyme^®^ (Novozymes A/S, Bagsvaerd, Denmark) were added, and the mixture was incubated at 50 °C for 3 h. The reaction mixture was centrifuged (4000× *g*, 10 min, 4 °C), and the supernatant was filtered under suction. To inactivate the enzymes, the filtrate was heated at ≥95 °C for 1 h, cooled, centrifuged again (4000× *g*, 10 min, 4 °C), and refiltered. The final supernatant was used as buckwheat flour hydrolysate.

### 2.3. Purification of Buckwheat Flour Hydrolysate

The hydrolysate was subjected to reverse-phase column chromatography using an ODS (octadecylsilyl) column (Cosmosil 140 C18; Nacalai Tesque, Kyoto, Japan) pre-equilibrated with distilled water. Elution was performed sequentially using distilled water (Fr. 1), 20% aqueous methanol (Fr. 2), 40% aqueous methanol (Fr. 3), and 100% methanol (Fr. 4). Fr. 1 was freeze-dried, whereas Fr. 2–Fr. 4 were evaporated. Yields of 4.915 g (Fr. 1), 193 mg (Fr. 2), 49 mg (Fr. 3), and 42 mg (Fr. 4) were obtained.

Fr. 2, which showed DPP-4 inhibitory activity, was dissolved in 5 mL of distilled water. Acetonitrile was added to adjust the final concentration to 95% (*v*/*v*) and the mixture was vortexed thoroughly. The solution was centrifuged (15,000× *g* for 5 min at 4 °C), the precipitate was dried under nitrogen to yield Fr. 2-1, and the supernatant was concentrated under reduced pressure and lyophilized to obtain Fr. 2-2. The yields were as follows: 142 mg (Fr. 2-1) and 50 mg (Fr. 2-2).

The active Fr. 2-2 was further purified by reverse-phase HPLC (high-performance liquid chromatography) using an Alliance e2695 system (Waters, Milford, MA, USA) equipped with an XSelect Peptide CSH C18 column (130 Å, 3.5 µm, 4.6 × 150 mm; Waters). The mobile phase comprised solvent A (150 mM ammonium bicarbonate–carbonate buffer, pH 8.0) and solvent B (methanol/acetonitrile, 50:50, *v*/*v*). The flow rate was set at 0.8 mL/min, and detection was performed at 215 nm. The gradient program was as follows: 0–3 min, 3% B; 3–60 min, a linear gradient from 3% to 100% B. Fractions were collected every 5 min from 0 to 50 min and dried under a nitrogen stream. Fr. 2-2 was separated into ten subfractions (Fr. 2-2-1 through Fr. 2-2-10).

Fr. 2-2-7 was purified using an Alliance e2695 system (Waters) equipped with a Superdex 30 Increase 10/300 GL column (9 µm; Cytiva, Marlborough, MA, USA). The mobile phase comprised solvent A (150 mM ammonium bicarbonate–carbonate buffer, pH 8.0) and solvent B (methanol/acetonitrile, 50:50 *v*/*v*) with a constant 20% B composition. The flow rate was 0.3 mL/min, and UV detection was performed at 215 nm. Fractions were collected every 5 min from 45 to 80 min. A highly active fraction eluting between 50 and 55 min was subjected to liquid chromatography–tandem mass spectrometry (LC–MS/MS) analysis.

Fr. 2-2-8 was further purified by HPLC using an Alliance e2695 system (Waters, Milford, MA, USA) equipped with an XSelect Peptide CSH C18 column (130 Å, 3.5 µm, 4.6 × 150 mm; Waters). The mobile phase consisted of solvent A (0.1% trifluoroacetic acid in water) and solvent B (methanol/acetonitrile/trifluoroacetic acid, 50:50:0.1 *v*/*v*/*v*). The flow rate was 0.8 mL/min, and detection was performed at 215 nm. The gradient program was as follows: 0–5 min, 25% B; 5–30 min, linear gradient 25–40% B; 30–35 min, 40% B; 35–45 min, linear gradient 40–100% B. The active fraction eluting between 11.5–14 min was subjected to LC–MS/MS analysis.

### 2.4. Identification of DPP-4 Inhibitory Peptides Using LC–MS/MS Analysis

A liquid chromatography–tandem mass spectrometry (LC-MS/MS) analysis of the DPP-4 inhibitory peptide in Fr. 2-2-7 was performed using a quadrupole time-of-flight tandem mass spectrometer (Q-TOF MS) (X500R, SCIEX, Concord, ON, Canada) coupled with an ultrahigh-performance liquid chromatography instrument (Nexera; Shimadzu, Kyoto, Japan) equipped with a reverse-phase column (CORTECS UPLC phenyl, 1.6 μm, 2.1 × 75 mm). The samples were eluted using solvent A (0.05% acetic acid in water) and solvent B (0.05% acetic acid in acetonitrile) with a gradient from 2% B to 98% B over 4.5 min at a flow rate of 0.3 mL/min. The Q-TOF MS conditions were as follows: positive-ion mode, ion spray voltage 5500 V, declustering potential 80 V, and collision energy 5 V. The MS/MS conditions were as follows: positive-ion mode, declustering potential 90 V, collision energy 15 V, and precursor ion *m*/*z* 342.2.

An analysis of the DPP-4 inhibitory peptides in the Fr. 2-2-8 was performed using an EASY-nLC1000 instrument (Thermo Scientific, San Jose, CA, USA). UHPLC separation was conducted using a NTCC-360/75-3-125 C18 column (3 mm, 0.075 mm × 120 mm; Nikkyo Technos, Tokyo, Japan) at 35 °C. The mobile phase comprised solvent A (0.1% formic acid in water) and solvent B (0.1% formic acid in acetonitrile). The gradient was as follows: 0–1 min, 0–5% B; 1–21 min, 5–35% B; 21–23 min, 35–90% B; 23–40 min, 90% B. The flow rate was set at 300 nL/min. An Orbitrap Elite^TM^ hybrid ion trap-Orbitrap mass spectrometer (Thermo Scientific, Waltham, MA, USA) combining Fourier transform and ion trap tandem MS was employed, and full MS scans were obtained in the Orbitrap to conduct accurate mass measurements. The selected precursor ions were subjected to collision-induced dissociation (CID) in an ion trap for fragment analysis and peptide structure elucidation.

### 2.5. Determination of Amino Acid Sequences of Active Peptides Using LC-MS/MS

An LC–MS/MS analysis of the tripeptide was conducted using a Q-TOF MS spectrometer (X500R; AB SCIEX, Concord, ON, Canada) with UHPLC (Nexera; Shimadzu, Kyoto, Japan) and a CORTECS UPLC C18 column (1.6 μm, 2.1 × 75 mm; Waters). The samples were eluted using solvent A (0.05% acetic acid in water) and solvent B (0.05% acetic acid in acetonitrile). The gradients were as follows: 0–1 min, 2% B; 1–2 min, 2–32% B; 2–12 min, 32% B. The flow rate was 0.3 mL/min. The MS/MS conditions were as follows: positive-ion mode, declustering potential of 90 V, and collision energy of 15 V. The precursor ions of peptides with molecular weights of 341 and 414 were set to *m*/*z* 342.2 and 415.1, respectively.

### 2.6. Evaluation of DPP-4 Inhibitory Activities of Fr. 2-2, Ile-Pro-Leu, and Ile-Pro-Trp After In Vitro Digestion

Pepsin and pancreatin (Fujifilm Wako Pure Chemical Corporation, Osaka, Japan) were dissolved in H_2_O (1 mg/mL) immediately before use. Fr. 2 was dissolved in H_2_O (3.0 mg/mL) and the pH was adjusted to 2. Hydrolysis was initiated by adding 1 mL of pepsin solution (enzyme/substrate = 1:25) and stopped after 1 h by adjusting the pH to 8. The pancreatin solution was then added to the pepsin hydrolysate (enzyme: substrate = 1:25). After 2 h, the reaction was terminated by boiling the mixture for 10 min. Control solutions were prepared identically without enzymes. Ile-Pro-Leu and Ile-Pro-Trp were dissolved in 50 mM Tris-HCl buffer (pH 7.5) at 1 mM and same procedure was applied. The resulting samples were used for DPP-4 inhibitory assays to evaluate the effects of in vitro digestion.

### 2.7. Oral Glucose Tolerance Test (OGTT) Using DPP-4 Inhibitory Fr. 2 in Rats

Male Sprague–Dawley rats (SD; 7 weeks old) were purchased from Japan SLC Inc. (Hamamatsu, Shizuoka, Japan). Animals were maintained in an air-conditioned room (25 °C) with a 12 h light/dark cycle. After a 7-day acclimation period with ad libitum access to standard feed (CRF-1; Oriental Yeast Co., Ltd., Tokyo, Japan) and water, the rats were fasted for 15 h. The control rats (*n* = 6) were orally administered with 2.5 mL of 40% glucose solution. Blood samples were collected from the tail vein at 0, 30, 60, 90, and 120 min, and glucose levels were measured using a glucose meter (Medisafé fit; Terumo Corporation, Tokyo, Japan). In the treatment group (*n* = 6), Fr. 2 dissolved in physiological saline, together with 40% glucose (*w*/*v*), was orally administered, and blood glucose was measured as in the control group. All animal experiments were conducted according to the guidelines of the Tokyo University of Agriculture Experimentation Committee (approval no. 240016, approved date on 20 August 2024).

### 2.8. Standard Peptides

Synthetic standard peptides (Ile-Pro-Leu, Ile-Pro-Ile, Leu-Pro-Leu, Leu-Pro-Ile, Ile-Pro-Trp, and Leu-Pro-Trp) were purchased from Scrum, Inc. (Tokyo, Japan).

## 3. Results and Discussion

### 3.1. DPP-4 Inhibitory Activity of Buckwheat Flour Hydrolysate

The DPP-4 inhibitory activity of buckwheat flour hydrolysate prepared with Alkalase^®^ and Flavorzyme^®^ was 60.8 ± 1.6% at a concentration of 10 mg/mL, whereas that of the control prepared by the same procedure without hydrolysis was 8.3 ± 1.2% at the same concentration. These results indicate that DPP-4 inhibitory peptides are present in the buckwheat flour hydrolysate.

**Figure 1:**
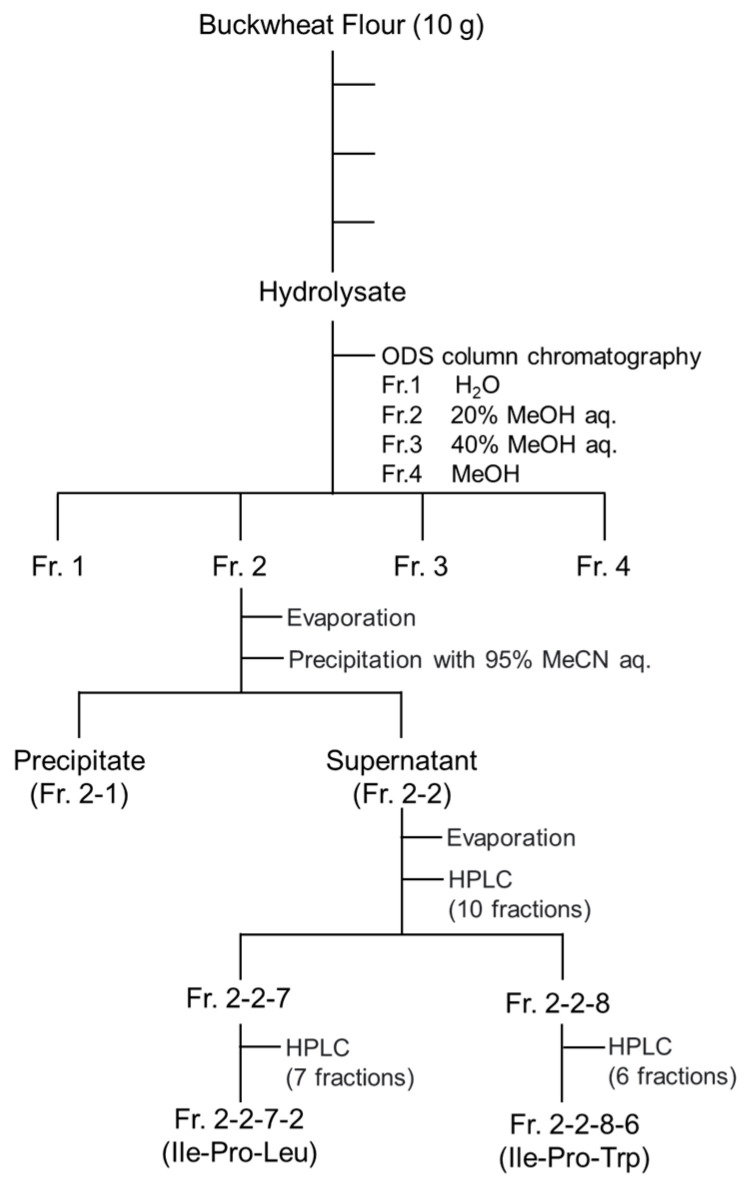
Separation of DPP-4 inhibitory peptides from buckwheat flour.

The hydrolysate was subjected to ODS column chromatography and sequentially eluted with H_2_O and 20% aqueous methanol, 40% aqueous methanol, and methanol, yielding fractions Fr. 1, Fr. 2, Fr. 3, and Fr. 4 (Figure 1). Among these, Fr. 2 exhibited the highest activity with an IC_50_ of 1.67 ± 0.11 mg/mL (Table 1). Since Fr. 1 and Fr. 4 showed markedly lower DPP-4 inhibitory activity relative to Fr. 2 and Fr. 3, we considered that determining the IC_50_ values for Fr. 1 and Fr.4 would not be meaningful within the scope of this study; therefore, the IC_50_ measurements were not conducted for Fr. 1 and Fr.4. The separation scheme for DPP-4 inhibitory peptides is provided in Figure 1.

### 3.2. Separation of DPP-4 Inhibitory Peptides in Fr. 2

**Figure 2:**
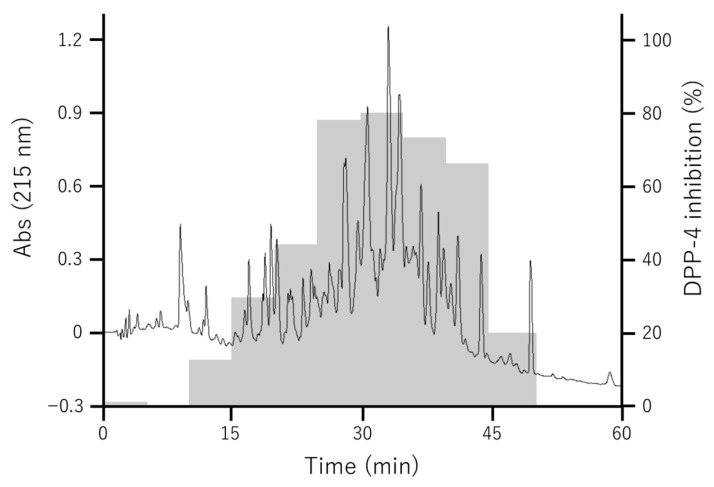
HPLC chromatogram of Fr. 2-2 with DPP-4 inhibitory activity. Fr. 2-2 was analyzed using an HPLC system equipped with a C18 column. The mobile phase consisted of solvent A (150 mM ammonium bicarbonate–carbonate buffer, pH 8.0) and solvent B (methanol/acetonitrile, 50:50, *v*/*v*). The flow rate was 0.8 mL/min, and detection was performed at 215 nm. Gradient elution was used to separate Fr. 2-2 into ten subfractions at 5-min intervals, and these were designated Fr. 2-2-1 through Fr. 2-2-10. The gray bars show the DPP-4 inhibitory activity of each fraction.

After evaporation, Fr. 2 was suspended in 95% aqueous acetonitrile to obtain a precipitate (Fr. 2-1) and a supernatant (Fr. 2-2). The DPP-4 inhibitory activities at 3 mg/mL were 60.3 ± 5.1% for Fr. 2-1 and 75.7 ± 8.2% for Fr. 2-2. As precipitation in 95% acetonitrile enriches less polar components, it was likely that the active peptides would have relatively low polarity. Fr. 2-2 was then subjected to reverse-phase HPLC, and fractions were collected at 5-min intervals to yield 10 fractions (Fr. 2-2-1 to Fr. 2-2-10) (Figure 2). Fr. 2-2-6 (25–30 min), Fr. 2-2-7 (30–35 min), and Fr. 2-2-8 (35–40 min) showed > 70% DPP-4 inhibitory activity at a concentration of 10 mg/mL (Table S1).

Because Fr. 2-2-7 showed relatively higher activity, it was then further separated by HPLC using a gel filtration column to obtain seven fractions (Fr. 2-2-7-1 to Fr. 2-2-7-7) (Figure S1). Fr. 2-2-7-2 showed the highest DPP-4 inhibitory activity (Table S2), and it was subjected to LC–MS/MS for peptide identification.

Since Fr. 2-2-8 displayed notable activity, it was further purified using reverse-phase HPLC to obtain six fractions (Fr. 2-2-8-1 to Fr. 2-2-8-6) (Figure S2). Fr. 2-2-8-6 showed the highest DPP-4 inhibitory activity (Table S3) and the peptide was then identified using LC–MS/MS.

### 3.3. Identification of Ile-Pro-Leu in Fr. 2-2-7-2

**Figure 3:**
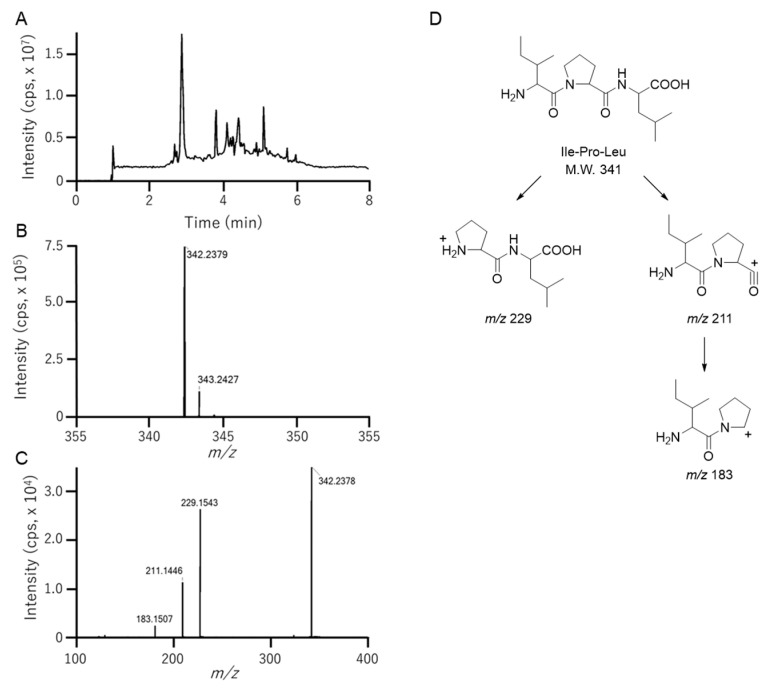
LC-MS analysis of Fr. 2-2-7-2 with DPP4 inhibitory activity. LC–MS/MS analysis was performed using a Q-TOF MS spectrometer coupled to a UPLC system equipped with a phenyl column. Samples were eluted using a gradient program with solvent A (0.05% acetic acid in water) and solvent B (0.05% acetic acid in acetonitrile). (**A**) Total ion chromatogram of Fr. 2-2-7-2. (**B**) Full scan MS spectrum of Fr. 2-2-7-2. (**C**) MS/MS spectrum derived from the precursor ion peak at *m*/*z* 342.2. (**D**) Fragmentation of Ile-Pro-Leu in LC-MS/MS. In LC-MS/MS analysis, exchanging leucine and isoleucine does not allow them to be distinguished by their mass spectra.

The LC–MS analysis of Fr. 2-2-7-2 revealed a major peak at *m*/*z* 342.2378 [M + H]^+^ (Figure 3), corresponding to C_17_H_31_N_3_O_4_ (*m*/*z* 342.2387 [M + H]^+^ calculated for C_17_H_32_N_3_O_4_). LC–MS/MS fragmentation of the *m*/*z* 342.2387 precursor indicated a tripeptide with a central proline and leucine or isoleucine flanking it, that is, Ile-Pro-Ile, Ile-Pro-Leu, Leu-Pro-Ile, or Leu-Pro-Leu (Figure 3). Ile-Pro-Ile is a potent DPP-4 inhibitor and is also known as diprotin A. This tripeptide was not detected in the food protein-derived hydrolysates. To determine whether the tripeptide found in this fraction was Ile-Pro-Ile, we attempted to identify it through LC-MS/MS analysis. As Leu and Ile are isobaric, the retention times of the synthetic standards of the four candidates were compared by LC-MS/MS (Figure S3). The retention time of the *m*/*z* 342.23 [M + H]^+^ peak in Fr. 2-2-7-2 matched that of Ile-Pro-Leu, but not the other tripeptides, identifying the peptide as Ile-Pro-Leu, which was previously reported as a DPP-4 inhibitory peptide in salmon milt [17]. To our knowledge, this is the first study to identify this peptide in a plant-derived protein hydrolysate. The IC_50_ of synthetic Ile-Pro-Leu in this study was 20.3 μM, whereas that of synthetic Ile-Pro-Ile was 2.3 μM. A minor variation in the side-chain structure of the third amino acid from the N-terminus of the tripeptides leads to an approximate 9-fold difference in the inhibitory activity, indicating that the side-chain structure of the third amino acid of Ile-Pro-Ile is crucial for inhibiting DPP-4 activity.

### 3.4. Identification of Ile-Pro-Trp in Fr. 2-2-8-6

**Figure 4:**
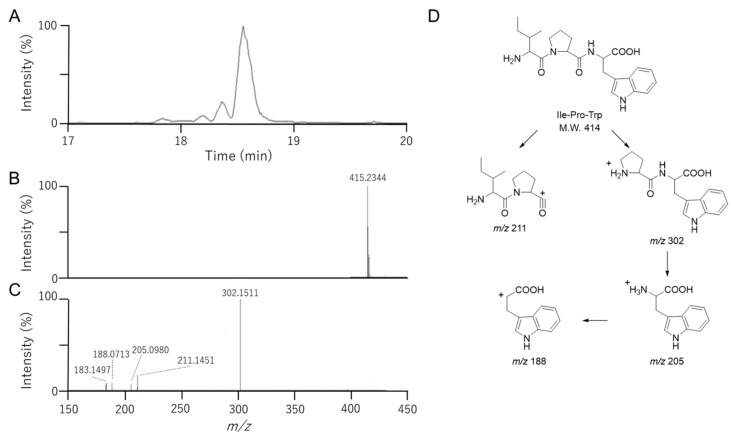
LC-MS analysis of Fr. 2-2-8-6 with DPP4 inhibitory activity. Samples were analyzed using a hybrid ion trap–Orbitrap mass spectrometer combining Fourier transform mass spectrometry (FT-MS) and ion trap tandem mass spectrometry (IT-MS/MS). HPLC separation was performed using an ODS column with a gradient of 0.1% formic acid in water and 0.1% formic acid in acetonitrile. (**A**) Total ion chromatogram of Fr. 2-2-8-6. (**B**) Full scan MS spectrum of Fr. 2-2-8-6. (**C**) MS/MS spectrum derived from the precursor ion peak at *m*/*z* 415.2. (**D**) Fragmentation analysis of Ile-Pro-Trp in LC-MS/MS. In LC-MS/MS analysis, exchanging leucine and isoleucine does not allow them to be distinguished by their mass spectra.

LC–MS/MS of Fr. 2-2-8-6 exhibited a major peak at *m*/*z* 415.2344 [M + H]^+^ (Figure 4), corresponding to C_22_H_30_N_4_O_4_ (*m*/*z* 415.2345 [M + H]^+^ calculated for C_22_H_31_N_4_O_4_). Fragmentation of the *m*/*z* 415.2 precursor indicated a tripeptide with leucine or isoleucine as the first residue, followed by proline and tryptophan (Figure 4), suggesting the presence of Ile-Pro-Trp or Leu-Pro-Trp. The LC-MS/MS comparison of the synthetic standards (Figure S4) showed that the retention time of Fr. 2-2-8-6 (*m*/*z* 415.2344 [M + H]^+^) matched that of Ile-Pro-Trp, identifying it as an active peptide. Although Ile-Pro-Trp has been chemically synthesized previously [18], there have been no reports of its purification from food protein hydrolysates. In the present study, the IC_50_ value of synthetic Ile-Pro-Trp was 6.2 μM; therefore, Ile-Pro-Trp showed considerable activity, despite being about one-third that of Ile-Pro-Ile. A previous study reported that Ile-Pro-Trp exhibits only weak DPP-4 inhibitory activity [18]; therefore, the DPP-4 inhibitory activity observed in this study are not fully consistent with the earlier findings. One possible explanation for this discrepancy is the differences between the assay conditions used to evaluate DPP-4 activity in the two studies. However, given that Val-Pro-Trp exhibits strong DPP-4 inhibitory activity [18], the observation that Val-Pro-Trp displays strong activity suggests consistency with our present finding that Ile-Pro-Trp also exhibits strong activity.

### 3.5. In Vitro Digestion of Peptides, Fr. 2, Ile-Pro-Leu and Ile-Pro-Trp

Because the peptides in Fr. 2 may be degraded in the stomach and intestines and thus lose their activity in vivo, we evaluated the effects of gastric and intestinal digestive enzymes on the DPP-4 inhibitory activity of Fr. 2. The residual DPP-4 inhibitory activity of Fr. 2 (3 mg/mL) after enzymatic treatments with pepsin and pancreatin was 101.7 ± 5.7%, showing no significant difference compared to Fr. 2 without enzymatic treatment. Furthermore, the residual DPP-4 inhibitory activity after the enzymatic treatment was evaluated for Ile-Pro-Leu and Ile-Pro-Trp at 1 mM, which were the active peptides identified in buckwheat hydrolysate. The residual DPP-4 inhibitory activities of Ile-Pro-Leu and Ile-Pro-Trp were 100.3 ± 5.1% and 99.4 ± 0.7%, respectively, indicating no significant difference from the peptides without enzymatic treatments. Accordingly, Fr. 2 and the active peptides identified in this study, Ile-Pro-Leu and Ile-Pro-Trp, exhibited minimal susceptibility to digestive enzymes. Although purified enzymes from swine were used in this study, and the same results cannot be directly assumed in humans, our findings indicate that porcine digestive enzymes did not significantly reduce the DPP-4 inhibitory activity of these peptides. These peptides may reach the intestinal tract intact and retain their bioactivity; however, this remains speculative and requires confirmation in humans.

### 3.6. OGTT Using DPP-4 Inhibitory Fr. 2 in Rats

**Figure 5:**
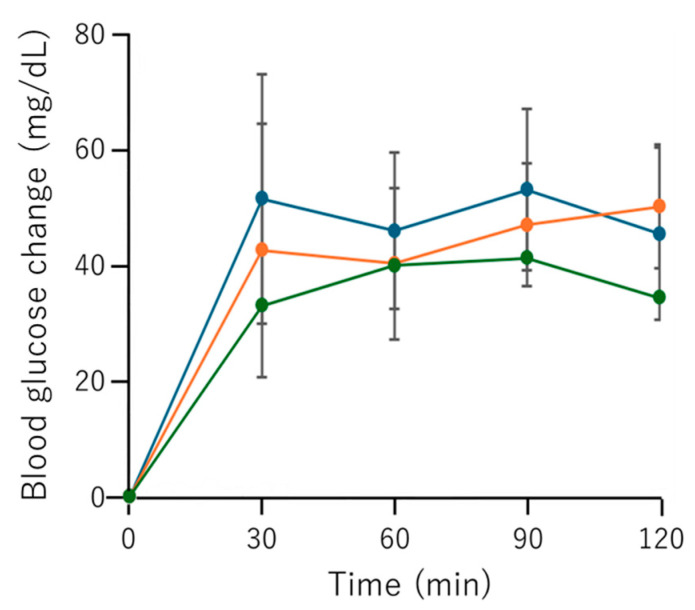
Blood glucose changes in the OGTT of rats administered Fr. 2. Data are expressed as the mean ± S.D. (*n* = 6). Statistical significance was determined using Dunnett’s test. The blue, orange, and green lines indicate the control group and groups administered 300 mg/kg and 600 mg/kg of Fr.2, respectively. Comparison of the control (blue) and 600 mg/kg group (green) at 30 min post-administration yielded *p* = 0.07.

To investigate the blood glucose-lowering effects of Fr. 2, the OGTT was performed in SD rats. Briefly, 1.0 g of glucose and Fr. 2 at 300 mg/kg and 600 mg/kg were administered orally, and blood glucose levels were measured at 0, 30, 60, 90, and 120 min (Figure 5). At 300 mg/kg, the differences between the results of Fr. 2 and the control were not substantially different at any time point. Fr. 2 administered at 600 mg/kg indicated a tendency to suppress glucose elevation at 30 min, but the difference was not statistically significant (*p* = 0.07). These findings suggest that Fr. 2 may have the potential to attenuate elevated blood glucose levels in SD rats. However, long-term administration studies and detailed analyses of blood glucose fluctuations at different time points would be necessary to further explore the possible usefulness of buckwheat flour hydrolysates.

## 4. Conclusions

This study identified two DPP-4 inhibitory peptides, Ile-Pro-Leu and Ile-Pro-Trp, from buckwheat flour hydrolysate. Ile-Pro-Trp is reported here for the first time as a food protein-derived peptide exhibiting potent DPP-4 inhibitory activity, while Ile-Pro-Leu was confirmed as a plant-derived DPP-4 inhibitor. Both peptides demonstrated clear activity, indicating that buckwheat proteins can serve as a source of bioactive peptides with glucose-regulating potential. Furthermore, oral administration of a peptide-enriched fraction derived from buckwheat suggested modest attenuation of postprandial blood glucose elevation in rats. These findings indicate the potential application of the identified peptides and buckwheat hydrolysates as functional food ingredients aimed at supporting glycemic control.

## Figures and Tables

**Table 1:** DPP-4 inhibitory activity of fractions obtained by ODS column chromatography of hydrolyzed buckwheat flour.

Fraction	Weight (g)	DPP-4 Inhibition (%, 10 mg/mL)	IC_50_ (mg/mL)
Fr. 1	4.915	17.0 ± 1.65	n.d.
Fr. 2	0.193	91.5 ± 1.8	1.67 ± 0.11
Fr. 3	0.049	90.3 ± 3.9	2.14 ± 0.08
Fr. 4	0.042	41.6 ± 10.1	n.d.

These values represent the mean ± S.D. (*n* = 3). “n.d.” means “not determined”.

## Data Availability

The original contributions presented in this study are included in the article/Supplementary Material. Further inquiries can be directed to the corresponding author.

## Supplementary Materials

The following supporting information can be downloaded at: https://www.mdpi.com/article/10.3390/foods15010092/s1, Figure S1: HPLC chromatogram of Fr. 2-2-7 with DPP-4 inhibitory activity; Figure S2: HPLC chromatogram of Fr. 2-2-8 with DPP-4 inhibitory activity; Figure S3: Comparison of the retention time of the tripeptide in Fr. 2-2-7-2 with candidate peptides Leu-Pro-Leu, Leu-Pro-Ile, Ile-Pro-Leu, and Ile-Pro-Ile; Figure S4: Comparison of the retention time of the tripeptide in Fr. 2-2-7-2 with candidate peptides Ile-Pro-Trp and Leu-Pro-Ile; Table S1: DPP-4 inhibitory activity of Fr. 2-2-1 through F. 2-2-10; Table S2: DPP-4 inhibitory activity of Fr. 2-2-7-1 through Fr. 2-2-7-7; Table S3: DPP-4 inhibitory activity of Fr. 2-2-8-1 through Fr. 2-2-8-6.
