# Supplementary material for: Dipeptidyl Peptidase-4 Inhibitory Activity of Buckwheat Flour-Derived Peptides and Oral Glucose Tolerance Test of Buckwheat Flour Hydrolysates in Rats
Source: Foods. 2025 Dec 29;15(1):92. doi: 10.3390/foods15010092 (PMC12785546; doi:10.3390/foods15010092)
Supplement: Supplementary file 1 [file foods-15-00092-s001.zip › foods-4028878-supplementary.pdf]

Supplemental materials

Title

Dipeptidyl peptidase-4 inhibitory activity of buckwheat flour-derived peptides and oral glucose tolerance test of buckwheat flour hydrolysates in rats

Authors

Noe Mitsui[1], Kouji Shiono[1], Yoshiya Seto[2], Tadasu Furusho[3], Chika Saito[1], Kosaku Takahashi[1*]

Affiliations

[1]Faculty of Applied Biosciences, Tokyo University of Agriculture

1-1-1 Sakuragaoka, Setagaya-ku, Tokyo 156-8502, Japan

[2]School of Agriculture, Meiji University

1-1-1 Higashi-Mita, Tama-ku, Kawasaki, Kanagawa 214-8571, Japan

[3]Faculty of International Agriculture Food Studies, Tokyo University of Agriculture

1-1-1 Sakuragaoka, Setagaya-ku, Tokyo 156-8502, Japan

*Correspondence: kt207119@nodai.ac.jp (K.T.)

Table S1. DPP-4 inhibitory activity of Fr. 2-2-1 through F. 2-2-10.

Fractions	DPP-4 inhibition (%)	Fractions	DPP-4 inhibition (%)
Fr. 2-2-1	3.6	Fr. 2-2-6	79.2
Fr. 2-2-2	0.9	Fr. 2-2-7	80.2
Fr. 2-2-3	12.5	Fr. 2-2-8	72.9
Fr. 2-2-4	33.3	Fr. 2-2-9	65.6
Fr. 2-2-5	44.0	Fr. 2-2-10	20.4

The values represent the means of three independent experiments. Fractions were collected every 5 min from 0 to 50 min.

Table S2. DPP-4 inhibitory activity of Fr. 2-2-7-1 through Fr. 2-2-7-7.

Fractions	DPP-4 inhibition (%)	Fractions	DPP-4 inhibition (%)
Fr. 2-2-7-1	0	Fr. 2-2-7-5	0
Fr. 2-2-7-2	32.5	Fr. 2-2-7-6	0
Fr. 2-2-7-3	23.0	Fr. 2-2-7-7	0
Fr. 2-2-7-4	16.2		

The values represent the means of three independent experiments. Fractions were collected every 5 min from 45 to 80 min.

Table S3. DPP-4 inhibitory activity of Fr. 2-2-8-1 through Fr. 2-2-8-6.

Fractions	Time (min)	DPP-4 inhibition (%)	Fractions	Time (min)	DPP-4 inhibition (%)
Fr. 2-2-8-1	1.8 to 4.5	1.2	Fr. 2-2-8-4	7.0 to 9.5	4.1
Fr. 2-2-8-2	4.5 to 5.5	6.8	Fr. 2-2-8-5	9.5 to 11.5	10.1
Fr. 2-2-8-3	5.5 to 7.0	8.3	Fr. 2-2-8-6	11.5 to14.0	45.0

The values represent the means of three independent experiments.

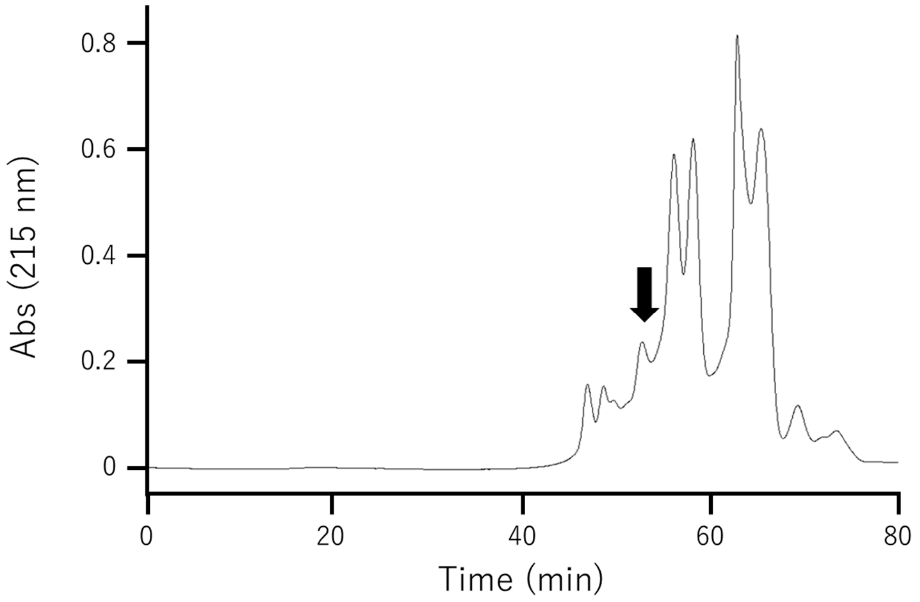

Fig. S1. HPLC chromatogram of Fr. 2-2-7 with DPP-4 inhibitory activity.

Fr. 2-2-7 was separated using HPLC equipped with a gel filtration chromatography column. The mobile phase consisted of solvent A (150 mM ammonium bicarbonate–carbonate buffer, pH 8.0) and solvent B (methanol/acetonitrile, 50:50 v/v), with a constant composition of 20% solvent B. The flow rate was 0.3 mL/min, and UV detection was performed at 215 nm. Fractions were collected at 5-minute intervals from 45 to 80 minutes. A highly active fraction eluting between 50 and 55 minutes was subjected to LC–MS/MS analysis. The arrow indicates the peak presumed to contain the active peptide.

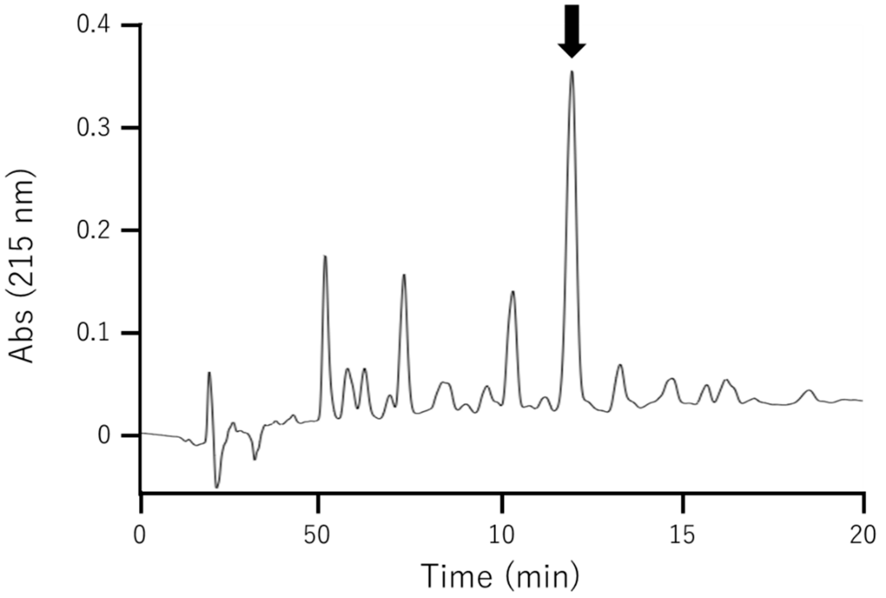

Fig. S2. HPLC chromatogram of Fr. 2-2-8 with DPP-4 inhibitory activity.

Fr. 2-2-8 was analyzed using an HPLC system equipped with a C18 column. The mobile phase consisted of solvent A (0.1% trifluoroacetic acid in water) and solvent B (methanol/acetonitrile/trifluoroacetic acid, 50:50:0.1 v/v/v). The flow rate was 0.8 mL/min, and UV detection was performed at 215 nm. An active fraction eluting between 11.5 and 14 minutes during gradient elution was purified and subjected to LC–MS/MS analysis. The arrow indicates the peak presumed to contain the active peptide.

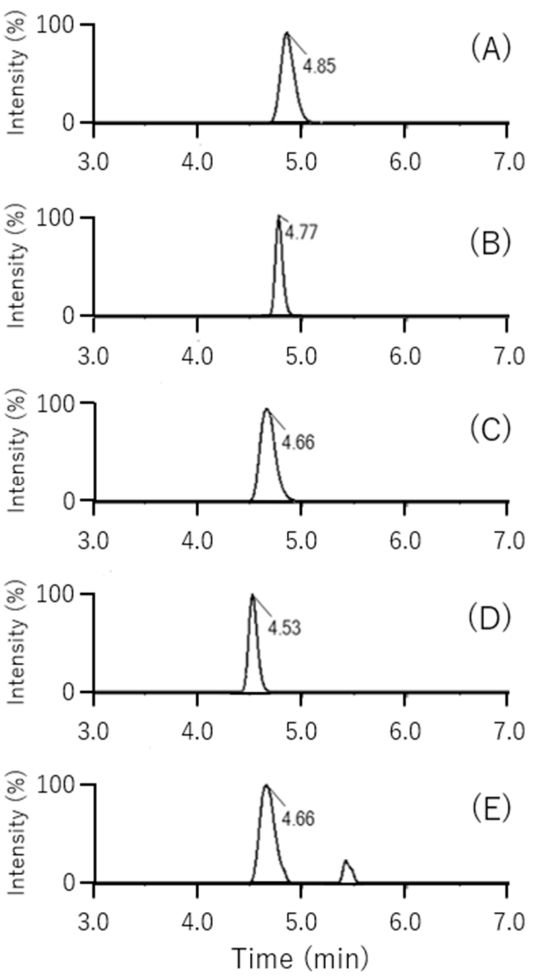

Figure S3. Comparison of the retention time of the tripeptide in Fr. 2-2-7-2 with candidate peptides Leu-Pro-Leu, Leu-Pro-Ile, Ile-Pro-Leu, and Ile-Pro-Ile.

LC–MS/MS analysis was carried out using a Q-TOF MS spectrometer and a UPLC equipped with an ODS column. The gradient program employed solvent A (0.05% acetic acid in water) and solvent B (0.05% acetic acid in acetonitrile). The precursor ion was set to *m/z* 342.2, and the product ion was monitored at *m/z* 227. (A): Leu-Pro-Leu; (B): Leu-Pro-Ile; (C): Ile-Pro-Leu; (D): Ile-Pro-Ile; (E): Fr. 2-2-7-2.

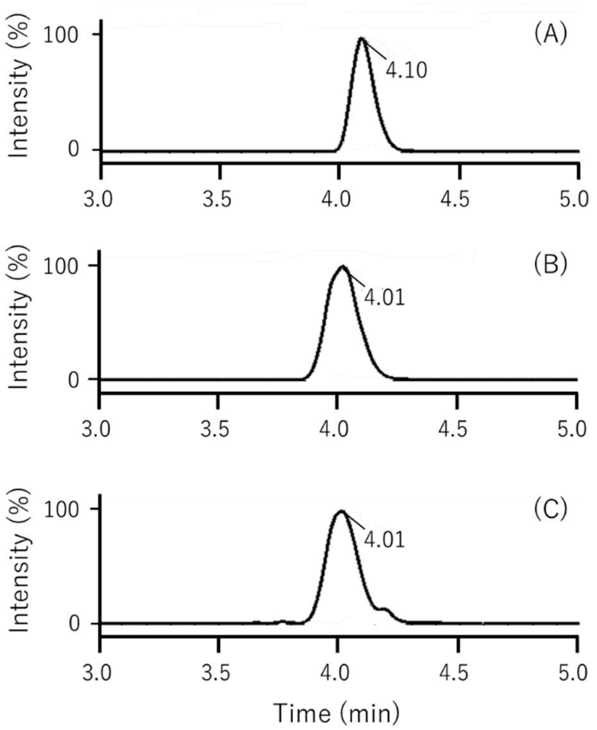

Figure S4. Comparison of the retention time of the tripeptide in Fr. 2-2-7-2 with candidate peptides Ile-Pro-Trp and Leu-Pro-Ile.

LC–MS/MS analysis was conducted using a Q-TOF MS spectrometer and a UPLC equipped with an ODS column. The gradient pro-gram consisted of solvent A (0.05% acetic acid in water) and solvent B (0.05% acetic acid in acetonitrile). The precursor ion was set to *m/z* 415.2, and the product ion was monitored at *m/z* 302. (A): Leu-Pro-Trp; (B): Ile-Pro-Trp; (C): Fr. 2-2-7-2.
